# Comparison of open and percutaneous A1 pulley release in pediatric trigger thumb: a retrospective cohort study

**DOI:** 10.1515/med-2025-1364

**Published:** 2025-12-19

**Authors:** Soner Kocak, Sabri Kerem Diril

**Affiliations:** Department of Orthopaedic and Traumatology, University of Health Sciences, Kanuni Sultan Suleyman Training and Research Hospital, Istanbul, Türkiye

**Keywords:** A1 pulley release, open surgery, outpatient surgery, pediatric trigger thumb, percutaneous technique, recurrence

## Abstract

**Objectives:**

Pediatric trigger thumb (PTT) is characterized by flexion deformity and interphalangeal joint locking caused by A1 pulley constriction. Open A1 pulley release is the standard surgical method, whereas percutaneous release under local anesthesia offers a minimally invasive outpatient alternative. This study compared the outcomes of these two techniques.

**Methods:**

A retrospective cohort of children aged 2–10 years undergoing A1 pulley release between 2012 and 2024 was analyzed. Patients were assigned to open release under general anesthesia or percutaneous release under local anesthesia. Demographics, operative details, complications, and outcomes were compared using appropriate statistical tests, with significance set at p<0.05.

**Results:**

Ninety-nine patients (107 thumbs) were included: 53 (58 thumbs) in the open group and 46 (49 thumbs) in the percutaneous group. Mean age at surgery was similar (4.33 ± 1.53 vs. 4.29 ± 1.70 years; p=0.781). Satisfactory results were achieved in 100 % of open and 85.7 % of percutaneous cases (p=0.003). Recurrence was 3.4 % and 8.2 %, respectively (p=0.409). No neurovascular or tendon injuries occurred; superficial infections were minor and limited to the open group.

**Conclusions:**

Both techniques are effective and safe. Open release remains the gold standard, while percutaneous release is a practical minimally invasive option in selected patients.

**Level of evidence:**

III, retrospective comparative study.

## Introduction

Pediatric trigger thumb (PTT) is a common disorder in children, characterized by flexion deformity and locking of the interphalangeal joint, typically caused by thickening or constriction of the A1 pulley, which impairs flexor pollicis longus tendon gliding and creates a dimensional mismatch between the tendon and pulley [1], 2]. The reported prevalence of PTT is up to 3.3 per 1,000 live births, and while its exact pathogenesis remains uncertain, it is generally attributed to a multifactorial interaction of congenital predisposition and acquired influences [3], [4], [5]. Management options include observation, splinting, stretching exercises, and surgical release, with surgery being indicated in cases refractory to conservative treatment, where it has consistently demonstrated safety and efficacy [6], [7], [8]. In clinical practice, conservative approaches are usually preferred in children younger than two years, whereas surgical A1 pulley release is commonly recommended for those older than two years [9], [10], [11].

A1 pulley release can be performed using either open or percutaneous techniques. Open release has been considered the standard approach, as it allows direct visualization of the pulley and adjacent neurovascular structures, thereby minimizing the risk of incomplete release and neurovascular injury. However, the need for hospitalization, operating room use, a surgical incision, and postoperative wound care are regarded as disadvantages. In contrast, percutaneous release does not require hospitalization or a surgical incision, and it can be performed under local anesthesia in an outpatient setting without the need for an operating room. Its minimally invasive nature and shorter operative time make it relatively simpler and faster, with little to no scar formation at the surgical site. Nonetheless, despite these theoretical advantages, concerns remain regarding incomplete release, iatrogenic injury, and recurrence [12], [13], [14].

Although both techniques have been well described in the literature, studies comparing their relative efficacy and safety have produced inconsistent findings, and no clear consensus has been established regarding the optimal approach. This gap underscores the need for further comparative research. Therefore, the aim of the present study is to evaluate and compare open and percutaneous A1 pulley release in pediatric trigger thumb, with particular emphasis on recurrence, complications, and both clinical and functional outcomes.

## Methods

This retrospective comparative study included pediatric patients who underwent surgery for trigger thumb at our institution between 2012 and 2024.

Patient data were extracted from hospital records. When medical documentation was incomplete or required clarification, structured telephone interviews were conducted with caregivers during routine outpatient visits to obtain complementary information. These interviews focused on verifying treatment adherence, follow-up compliance, and developmental milestones. Only patients with complete medical documentation who met the predefined inclusion criteria were enrolled in the study. Demographic variables included age, sex, laterality, and socioeconomic status (SES). The study population represented a broad range of socioeconomic backgrounds.

The study included patients aged 2–10 years who were diagnosed with pediatric trigger thumb presenting with flexion contracture and/or interphalangeal joint locking, whose deformity persisted beyond the age of 2 despite failed conservative treatment, and who underwent primary A1 pulley release using either an open or percutaneous technique. Patients were excluded if they had a history of previous thumb surgery, major trauma or fracture involving the affected hand, associated congenital hand anomalies, neuromuscular disorders, or syndromic conditions. Additional exclusion criteria were loss to follow-up, insufficient postoperative data, or incomplete medical records that could not be verified through caregiver interviews.

The primary clinical findings in affected thumbs were fixed flexion contracture, the presence of a Notta’s nodule, and pain. Additional findings included loss of active extension and triggering or snapping phenomena. Medical records of patients diagnosed with trigger thumb were reviewed for age at presentation, age at onset of triggering, age at surgery, presence of a palpable nodule, and fixed flexion contracture.

Operative notes were examined for anesthesia type, surgical technique, timing of the procedure, flexor tendon condition, and details of the pulley release. All patients were followed for a minimum of 12 months. Outcomes and recurrence were evaluated both by the treating physicians during scheduled outpatient visits and by parents.

Postoperative assessments included evaluation of triggering, range of motion, persistence of flexion contracture, pain, scar formation, flexor tendon integrity, and complications such as hematoma, swelling, infection, sensory deficit, or neurovascular injury. Digital nerve function was tested on the ulnar and radial aspects of the thumb by assessing light touch and pinprick sensations and comparing them with adjacent digits. Additionally, the parental assessment during follow-up interviews provided a subjective measure of functional outcome related to daily activities.

Satisfactory postoperative outcome was defined as full range of motion with complete extension and absence of triggering. Residual triggering, incomplete extension, or reduced motion was classified as unsatisfactory. In bilateral cases, each thumb was analyzed as an independent unit. Recurrence was defined as reappearance of flexion contracture and/or interphalangeal joint locking during follow-up.

This study was conducted using a retrospective cohort design, and patients were divided into two groups. One surgeon performed open A1 pulley release under general anesthesia in the operating room, whereas the other surgeon carried out percutaneous release under local anesthesia in the outpatient clinic.

## Surgical techniques

### Open release

**Figure 1:**
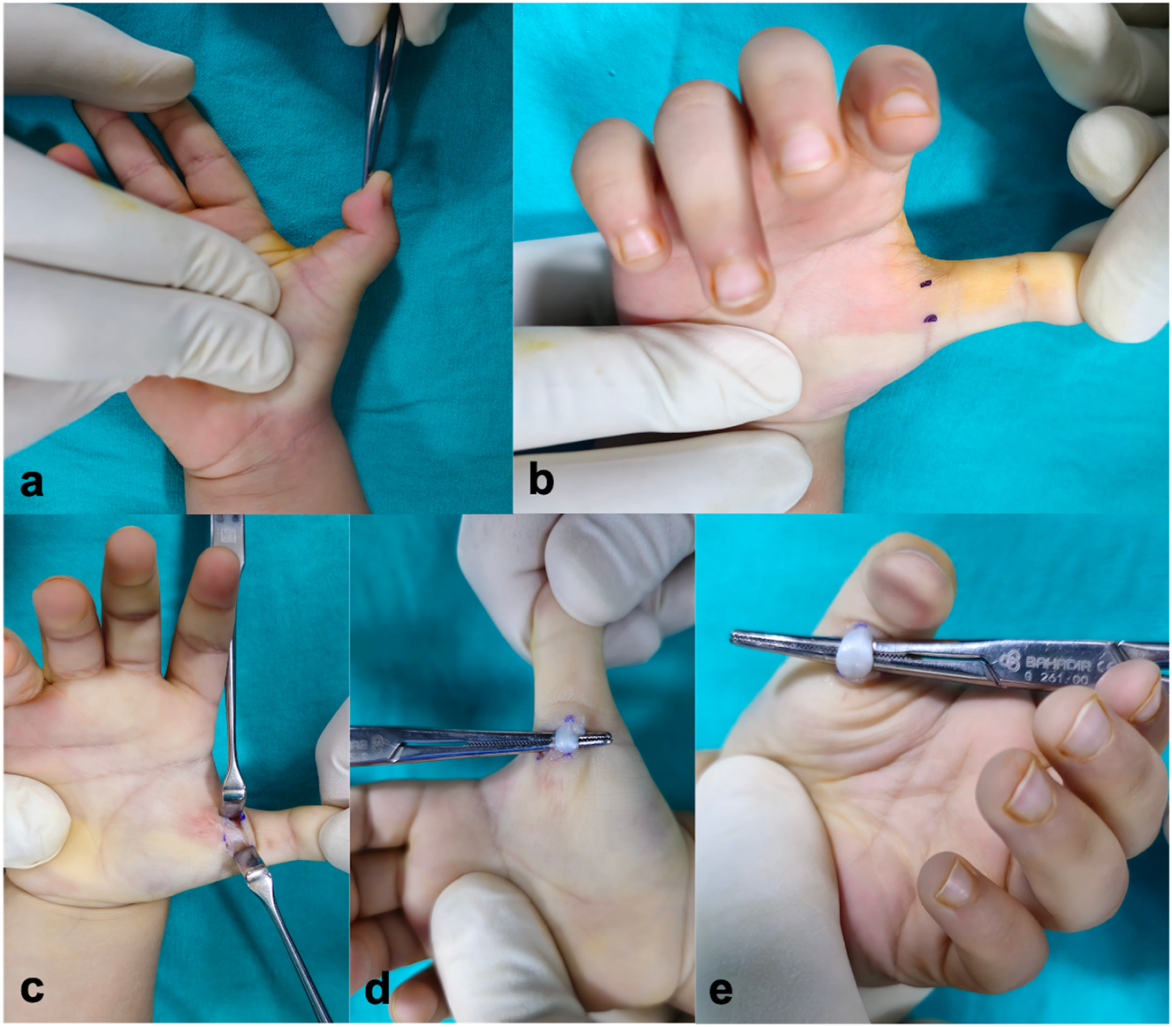
Open A1 pulley release. (a) Preoperative view showing fixed flexion contracture of the thumb. (b) Intraoperative marking of Notta’s nodule and the flexor tendon pathway. (c) Exposure of the A1 pulley achieved through appropriate retraction. (d) Passive thumb extension used to confirm complete tendon release following A1 pulley division. (e) Passive flexion of the interphalangeal joint to verify smooth tendon gliding after release.

Open A1 pulley release was performed under short-duration general anesthesia with tourniquet control. A transverse incision was made along the palmar crease overlying the first metacarpophalangeal joint. Dissection was carefully carried out to expose the flexor sheath while preserving the digital neurovascular bundles. The A1 pulley was identified under direct visualization and fully released. Flexor tendons and neurovascular structures were meticulously protected throughout the procedure. Intraoperatively, residual triggering was checked by passively flexing and fully extending the interphalangeal joint (Figure 1). The skin was closed with absorbable sutures. To prevent hematoma and swelling, gentle compression and a light dressing were applied for 10 min postoperatively. Patients were discharged the following day.

### Percutaneous release

**Figure 2:**
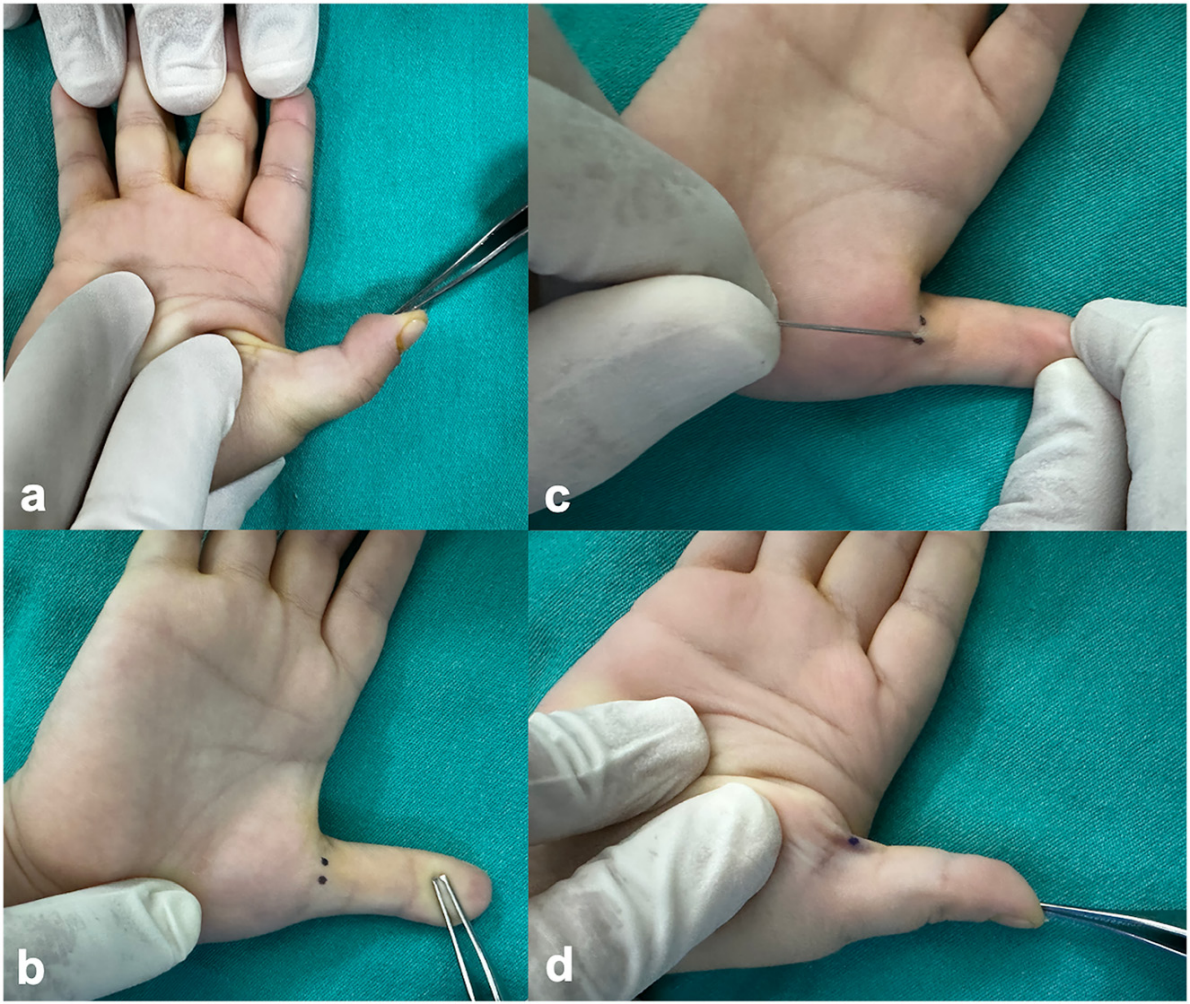
Percutaneous A1 pulley release. (a) Preoperative presentation of a fixed flexion contracture in the thumb. (b) Intraoperative identification of Notta’s nodule and the flexor tendon pathway, followed by marking and selection of the needle entry site. (c) Percutaneous division of the A1 pulley performed by inserting the needle just distal to the nodule and applying longitudinal sweeping movements. (d) Intraoperative assessment of tendon release through passive extension of the thumb.

All percutaneous releases were performed by the same surgeon under local anesthesia, without the use of a tourniquet. A digital nerve block was administered with 1 mL of 2 % lidocaine. With the thumb placed in abduction and extension, a 21-gauge hypodermic needle was introduced through the skin at the level of the A1 pulley, just distal to the nodule on the palmar surface. The needle was positioned parallel to the flexor tendon and advanced 3–5 mm to penetrate it. Proper intratendinous positioning was confirmed by passive thumb motion. The needle was then slightly withdrawn and swept proximally to distally along the tendon axis while maintaining passive extension, thereby dividing the A1 pulley in a controlled manner. To minimize the risk of nerve injury, the entry point was selected at the metacarpophalangeal crease and kept close to the midline. Complete release was confirmed intraoperatively by restoration of full interphalangeal flexion and extension and disappearance of locking (Figure 2). All patients tolerated the percutaneous procedure well; no sedation was required, parents were allowed to remain with the child to reduce anxiety, and no adverse events related to intolerance or inadequate anesthesia occurred. No sutures were required. A light compressive dressing was applied for 10 min postoperatively, and all patients were discharged on the same day.

All patients received standard postoperative care. Early postoperative assessments focused on bleeding, swelling, and hematoma formation. A light compressive dressing was applied with the thumb in extension for one week, and nonsteroidal anti-inflammatory medication was prescribed as needed. Follow-up visits were scheduled at 1–2 weeks, 1 month, and at the final evaluation.

To minimize observer bias, postoperative assessments were conducted by an independent surgeon blinded to the treatment groups. Demographic variables, including age, sex, and laterality, were documented to ensure comparability between groups. Potential confounders – such as age at release, sex, and laterality – were adjusted for using post-hoc multivariate logistic regression analysis. Although randomization was not feasible due to the retrospective nature of the study, methodological rigor was preserved through comprehensive data collection and appropriate statistical adjustments to reduce bias.

The study protocol was approved by the institutional ethics committee, and informed consent was obtained from the parents or legal guardians of all participants.

### Statistical analysis

All statistical analyses were conducted using SPSS Statistics version 25.0. Normality of continuous variables was assessed using the Shapiro–Wilk test. Age and follow-up duration were analyzed as continuous variables. Age demonstrated normal distribution and was analyzed using the Student’s *t*-test, whereas follow-up duration, which did not demonstrate normality, was analyzed using the Mann–Whitney U test. Categorical variables including sex, laterality, recurrence, and complication rates were analyzed using the Chi-square test or Fisher’s exact test when expected cell counts were <5. Group comparisons for continuous variables were performed with either the Student’s *t*-test or the Mann–Whitney U test, depending on distributional assumptions, whereas categorical variables were analyzed using the Chi-square or Fisher’s exact test as appropriate. Statistical significance was defined as p<0.05.

## Results

A total of 99 patients (107 thumbs) were included in the study. The open release group comprised 53 patients (58 thumbs), while the percutaneous release group included 46 patients (49 thumbs). The mean follow-up period was 49 months (range, 12–68 months) for the open group and 38 months (range, 12–58 months) for the percutaneous group.

The sex distribution in the open group was 28 males (52.8 %) and 25 females (47.2 %), compared to 20 males (43.5 %) and 26 females (56.5 %) in the percutaneous group. There was no statistically significant difference between groups regarding sex distribution (p=0.467).

The mean age at the time of surgery was 4.33 ± 1.53 years (range, 2–8 years) in the open group and 4.29 ± 1.70 years (range, 2–9 years) in the percutaneous group, with no significant difference between groups (p=0.781).

Regarding laterality, 30 right (51.7 %) and 28 left (48.3 %) thumbs underwent surgery in the open group, while 24 right (49.0 %) and 25 left (51.0 %) thumbs were operated on in the percutaneous group. Side distribution was comparable, with no significant difference between groups (p=0.929) (Table 1).

**Table 1:** Demographic characteristics of patients.

	Open release	Percutaneous release	p-Value
Cases; Unilateral Bilateral	53 (100 %) 48 (90.6 %) 5 (9.4 %)	46 (100 %) 43 (93.5 %) 3 (6.5 %)	0.73
Sex; Male Female	28 (52.8 %) 25 (47.2 %)	20 (43.5 %) 26 (56.5 %)	0.467
Laterality (thumbs); Right Left	30 (51.7 %) 28 (48.3 %)	24 (49 %) 25 (51 %)	0.929
Age at surgery (years, mean ± SD, range)	4.33 ± 1.53 (2–8)	4.29 ± 1.70 (2–9)	0.781
Follow-up duration (month)	49 (range 12–68)	38 (range 12–58)	

All thumbs in the open release group achieved satisfactory outcomes (58/58; 100 %). In the percutaneous group, 42 thumbs (85.7 %) had satisfactory results, while 7 thumbs (14.3 %) were classified as unsatisfactory. The difference in outcomes between the two groups was statistically significant (p=0.003). Recurrence occurred in 2 thumbs (3.4 %) in the open group and in 4 thumbs (8.2 %) in the percutaneous group, with no statistically significant difference between groups (p=0.409) (Table 2).

**Table 2:** Surgical outcomes and recurrence.

	Open release (n=58)	Percutaneous release (n=49)	p-Value
Outcome (thumbs); Satisfactory Unsatisfactory	58 (100 %) 0 (0 %)	42 (85.7 %) 7 (14.3 %)	0.003
Recurrence	2 (3.4 %)	4 (8.2 %)	0.409

Superficial postoperative infection was observed in two open-release cases, both of which resolved with oral antibiotics; no recurrences were noted in these patients. No cases of nerve injury, flexor tendon injury, sensory deficit, hyperextension deformity of the metacarpophalangeal joint, or postoperative motion restriction were encountered during follow-up. All patients maintained normal grip function in daily activities, and those with satisfactory surgical results achieved grip strength comparable to the contralateral thumb.

Patients with unsatisfactory outcomes underwent secondary open surgical release. Similarly, in both groups, cases with recurrence during follow-up were managed with revision open release.

## Discussion

This retrospective comparative study evaluated the clinical and functional outcomes of percutaneous A1 pulley release performed under local anesthesia in the outpatient setting vs. open release under general anesthesia in the operating room for the treatment of pediatric trigger thumb. Both techniques were found to be effective and safe, demonstrating high success rates and low complication rates. However, notable differences emerged between the two approaches in terms of success rate, recurrence, procedural logistics, and perioperative considerations.

Previous studies have established open A1 pulley release as the standard surgical approach in most centers, reporting excellent success rates with minimal recurrence and low complication rates, which is consistent with our findings [2], 13], 15], 16]. In our cohort, open release achieved a 100 % satisfactory rate, with only two cases of superficial infection and no neurovascular or tendon injury. The recurrence rate of 3.4 % aligns with previously reported ranges [13], 17], 18]. These findings are further supported by a recent long-term study evaluating 67 pediatric trigger thumbs with a minimum follow-up of 10 years, in which 94 % of patients achieved full interphalangeal extension, the median QuickDASH score was 0, overall satisfaction was high (84 % reporting the maximum score), and no cases of residual triggering or revision surgery were observed [19].

Percutaneous release performed under local anesthesia yielded a satisfactory outcome in 85.7 % of cases without any major complications or neurovascular injuries. This finding is comparable to pediatric series that reported success rates between 92 % and 97 % for percutaneous techniques performed in outpatient settings [12], 20], 21]. However, Masquijo et al. cautioned against the routine use of percutaneous methods in children, citing incomplete pulley division and flexor tendon laceration as potential concerns [14]. In our study, although the recurrence rate in the percutaneous group (8.2 %) was slightly higher than in the open group, the difference was not statistically significant. Nevertheless, a systematic review by Sirithiantong et al. reported that percutaneous release increased the risk of recurrence 3.29-fold compared to open surgery, recommending open release as the preferred surgical option when intervention is required [22].

Clinically, both techniques are viable but serve different contexts. Open release allows direct visualization of the pulley and adjacent neurovascular structures, minimizing the risk of incomplete division and flexor tendon injury. Although recent pediatric anesthesia literature indicates that short-term general anesthesia does not significantly differ from awake regional anesthesia regarding long-term neurodevelopmental outcomes, parents often prefer to avoid general anesthesia when possible [23]. Conversely, percutaneous release offers distinct logistical advantages – it can be performed in the outpatient clinic without general anesthesia, hospitalization, or operating room requirements, while leaving minimal scarring. This makes it an attractive option for compliant patients with typical anatomy, particularly in resource-limited settings or for children in whom minimizing anesthetic exposure is desirable. Furthermore, a 2022 randomized controlled trial demonstrated that targeted local anesthetic infiltration prior to incision significantly reduced intraoperative general anesthetic requirements during pediatric trigger thumb release, supporting the feasibility and anesthetic-sparing advantages of outpatient percutaneous procedures in appropriately selected children [24].

From a healthcare systems perspective, outpatient percutaneous procedures may reduce hospital stay, surgical waiting times, and overall costs while maintaining favorable outcomes. Hand surgery literature supports the transition of minor procedures from operating rooms to outpatient clinics as a means to achieve substantial cost savings without compromising clinical efficacy [25], 26]. Such logistical and economic considerations are increasingly relevant in modern healthcare delivery. In a general sense, the elimination of operating room time, general anesthesia, and an overnight hospital stay indicates that the percutaneous procedure is substantially less resource-intensive and is likely associated with lower direct healthcare costs compared with the open procedure.

In our series, no postoperative stiffness, neurovascular or flexor tendon injuries occurred in either group, consistent with findings from recent large cohorts [12], 13]. Although the digital nerve course over the A1 pulley theoretically increases the risk of nerve injury during percutaneous release [14], careful technique with a midline entry point and controlled needle motion minimizes this risk. The recurrence rate remained low and was not statistically different between groups. We acknowledge that the difference in mean follow-up duration between the groups (49 months in the open group vs. 38 months in the percutaneous group) warrants consideration when interpreting recurrence rates. However, all recurrence events in our cohort were detected within the first 12 months after surgery, a period that was fully encompassed by the follow-up duration of both groups. Therefore, although the open group had a longer overall follow-up, this difference is unlikely to have substantially biased recurrence detection. In most cases, recurrence likely reflected incomplete pulley division rather than true regrowth, underscoring the importance of technical precision. These considerations are further supported by a 2024 systematic review and meta-analysis of 599 pediatric trigger thumbs, which reported that approximately 43.5 % of cases resolved spontaneously overall, with higher rates of resolution in cohorts followed for more than two years, underscoring the importance of follow-up duration when interpreting long-term outcomes and apparent recurrence rates after treatment [27].

This study has several limitations. Its retrospective design introduces a potential for selection bias; for instance, more complex or severe cases may have been directed toward open release. Although randomization was not performed, potential confounders such as age, sex, and laterality were adjusted for using multivariate analysis. Minor differences in follow-up duration between groups may have influenced recurrence detection. Moreover, standardized patient-reported outcome measures (PROMs) were not systematically collected. The assessment of functional outcome was therefore limited to parental reports of daily activities, general grip strength comparison, and objective range of motion findings, rather than utilizing validated instruments. This represents a notable limitation, as anatomical and mechanical results alone may not fully capture the patient’s functional experience or perceived treatment success. Finally, no cost analysis was performed, which could have provided valuable insights for healthcare policy and resource allocation.

## Conclusions

Both open and percutaneous A1 pulley release are effective and safe for the treatment of pediatric trigger thumb. Open release remains the gold standard, offering excellent outcomes with minimal recurrence, while percutaneous release provides comparable results in selected patients, with the additional advantages of outpatient feasibility, avoidance of general anesthesia, and lower associated healthcare resource utilization. With meticulous technique and appropriate patient selection, percutaneous release represents a practical minimally invasive alternative to open surgery. Future prospective, randomized multicenter studies with larger, stratified cohorts are warranted to further refine patient selection criteria and optimize treatment algorithms.
